# DNMT3B *in vitro* knocking-down is able to reverse embryonal rhabdomyosarcoma cell phenotype through inhibition of proliferation and induction of myogenic differentiation

**DOI:** 10.18632/oncotarget.12688

**Published:** 2016-10-15

**Authors:** Francesca Megiorni, Simona Camero, Simona Ceccarelli, Heather P. McDowell, Olga Mannarino, Francesco Marampon, Barry Pizer, Rajeev Shukla, Antonio Pizzuti, Cinzia Marchese, Anna Clerico, Carlo Dominici

**Affiliations:** ^1^ Department of Paediatrics and Infantile Neuropsychiatry, Sapienza University of Rome, Rome, Italy; ^2^ Department of Experimental Medicine, Sapienza University of Rome, Rome, Italy; ^3^ Department of Oncology, Alder Hey Children's NHS Foundation Trust, Liverpool, United Kingdom; ^4^ Department of Biotechnological and Applied Clinical Sciences, University of L'Aquila, L'Aquila, Italy; ^5^ Department of Perinatal and Paediatric Pathology, Alder Hey Children's NHS Foundation Trust, Liverpool, United Kingdom

**Keywords:** rhabdomyosarcoma, DNMT3B, RNA interference, myogenic differentiation, MEK/ERK signalling

## Abstract

Aberrant DNA methylation has been frequently observed in many human cancers, including rhabdomyosarcoma (RMS), the most common soft tissue sarcoma in children. To date, the expression and function of the *de novo* DNA methyltransferase (DNMT) 3B in RMS have not yet been investigated. Our study show for the first time a significant up-regulation of DNMT3B levels in 14 RMS tumour samples and 4 RMS cell lines in comparison to normal skeletal muscle. Transfection of RD and TE671 cells, two *in vitro* models of embryonal RMS (ERMS), with a synthetic DNMT3B siRNA decreased cell proliferation by arresting cell cycle at G1 phase, as demonstrated by the reduced expression of Cyclin B1, Cyclin D1 and Cyclin E2, and by the concomitant up-regulation of the checkpoint regulators p21 and p27. DNMT3B depletion also impaired RB phosphorylation status and decreased migratory capacity and clonogenic potential. Interestingly, DNMT3B knock-down was able to commit ERMS cells towards myogenic terminal differentiation, as confirmed by the acquisition of a myogenic-like phenotype and by the increased expression of the myogenic markers MYOD1, Myogenin and MyHC. Finally, inhibition of MEK/ERK signalling by U0126 resulted in a reduction of DNMT3B protein, giving evidence that DNMT3B is a down-stream molecule of this oncogenic pathway.

Taken together, our data indicate that altered expression of DNMT3B plays a key role in ERMS development since its silencing is able to reverse cell cancer phenotype by rescuing myogenic program. Epigenetic therapy, by targeting the DNA methylation machinery, may represent a novel therapeutic strategy against RMS.

## INTRODUCTION

Rhabdomyosarcoma (RMS) is the most common soft tissue sarcoma in childhood and adolescence, accounting for about 5% of malignant paediatric tumours [1]. The two main histological subtypes, alveolar RMS (ARMS) and embryonal RMS (ERMS), are associated with particular cytogenetic abnormalities and with different outcome [2]. ARMS is a high-grade malignancy and the majority of cases (90%) is characterized by either a t(2;13) or t(1;13) translocation, resulting in the expression of PAX3:FOXO1 or PAX7:FOXO1 fusion proteins [2, 3]. ERMS, the most common RMS type, is associated with a more favourable outcome and commonly exhibits 11p15 loss of heterozygosity [2] and/or mutations in components of the RAS pathway [4]. Presently, there is a growing evidence that epigenetic mechanisms play an important role in cancer development, with changes in DNA methylation being a crucial event in the control of gene expression [5]. The enzymatic addition of a methyl group at the carbon 5 position of cytosine, in the context of the sequence 5′ cytosine-guanosine (CpG), controls the expression of many tissue-specific genes [6–8], as well as several important cellular functions, such as X chromosome inactivation and genomic imprinting [9]. In mammalians, methylation of DNA is catalysed by the DNA methyltransferase (DNMT) enzymes 1, 3A and 3B. DNMT1 is required for the methylation maintenance by preferentially methylating the unmethylated strand of hemimethylated DNA during DNA replication [10, 11], whilst DNMT3A and DNMT3B are necessary for the establishment of *de novo* methylation of both strands during development [11]. Indeed, DNMT3A and DNMT3B are highly expressed in embryonic cells, where most new methylation events occur, and are down-regulated in differentiated cells [11, 12]. Over-expression of DNMTs has been described in several human tumour types, including lymphomas, liver, prostate, colorectal, breast, lung, pancreatic and endometrial cancer [13–18], and is generally associated with a more aggressive phenotype, indicating that DNMT1, 3A and 3B likely act as oncogenes [19]. Recent evidences suggest that a deregulation of the *de novo* DNMT enzymes contributes to the development of paediatric brain tumours [20]. Silencing of tumour suppressor genes (TSGs) by promoter CpG island hypermethylation seems to be the most probable mechanism involved in the process of carcinogenesis linked to epigenetic events. Indeed, transcriptional repression of different TSGs, such as p16/CDKN2A, RASSF1, MLH1, *etc*., has been extensively described in several malignancies and has been mainly linked to the aberrant quantity/activity of DNMT3 family enzymes [21, 22]. DNMTs inhibit gene expression by also recruiting transcriptionally repressive complexes, which include methyl-CpG binding domain (MBD) factors, polycomb group (PcG) proteins and repressor factors, to specific DNA sites [23–27]. Distinct patterns of DNA hypermethylation have been found in different types of cancers, confirming the importance to better understand the molecular mechanisms that mediate epigenetic phenomena. Because epigenetic alterations are reversible, DNMTs also represent attractive therapeutic targets since their inhibition might have the ability to overturn tumour cell phenotype [28–30]. Increased DNMT1 activity in RMS was previously reported [31] and DNA methylation signatures have been recently described in ARMS and ERMS cases [32], suggesting that aberrant DNA methylation may contribute to the development of RMS. Indeed, hypermethylated CpG islands have been identified in several genes involved in skeletal muscle development and differentiation in both RMS cell lines and primary tumour samples [32–34]. Consistently, treatment with 5-aza-2'-deoxycytidine (5-aza-dC), a general DNA demethylating agent, is able to suppress tumorigenicity and partially reactivate myogenic program in different RMS cell lines [34, 35]. Despite these evidences, the expression and the individual role of DNMT3B have not yet been characterised in this malignancy. Since different studies linked DNMT3B over-expression to gene-specific hypermethylation during cancer development and progression [36, 37], we investigated the DNMT3B function in RMS. Our study reveals that DNMT3B levels are significantly up-regulated in RMS tumours and cell lines in comparison with normal skeletal muscle (NSM). Notably, DNMT3B knock-down by RNA interference is able to significantly arrest cell proliferation and promote myogenic differentiation of ERMS cell lines. Indeed, DNMT3B-depleted cells changed their morphology, with a more elongated appearance, and expressed increased levels of the myogenic proteins MYOD1 (myoblast determination protein 1) and Myogenin as well as the terminal differentiation marker MyHC (myosin heavy chain). We also suggest that DNMT3B is a down-stream important molecule in the mitogen-activated protein kinase kinase/extracellular signal-regulated kinase (MEK/ERK) signalling pathway since treatment with U0126, a known inhibitor of ERK phosphorylation, drastically down-regulates DNMT3B protein levels in ERMS cells, this leading to growth arrest and terminal myogenic differentiation.

These findings underline how the dysregulation of DNA methylation is associated with RMS transformation and aggressiveness, and suggest that impairing DNMT3B activity may be a promising tool for treatment of RMS, especially the ERMS subtype.

## RESULTS

### DNMT3B quantification in RMS tumours and cell lines

**Figure 1:**
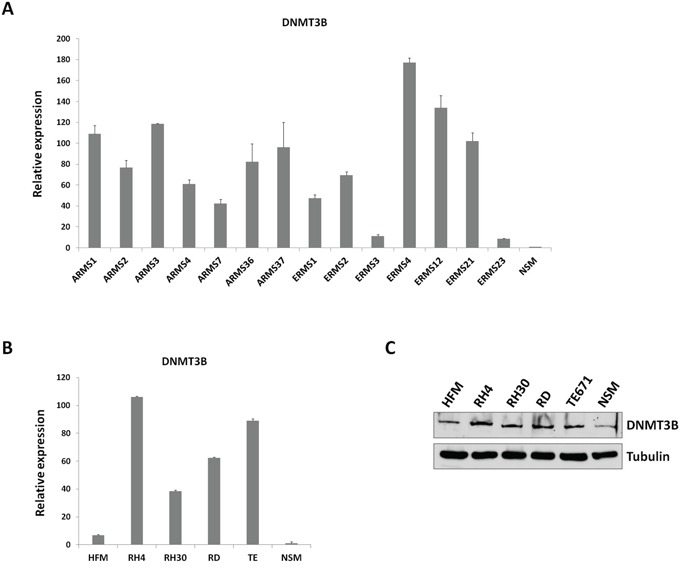
DNMT3B expression in RMS tumours and cell lines **A.** Quantitative real time PCR (Q-PCR) analysis of DNMT3B mRNA levels in 14 RMS primary tumours (7 ARMSs and 7 ERMSs), expressed as fold increase over NSM, arbitrarily set at 1. Transcript levels were normalized to GAPDH mRNA and error bars represent SD of two independent Q-PCR reactions, each performed in triplicate. **B.** Q-PCR of DNMT3B mRNA levels in ARMS (RH4 and RH30) and ERMS (RD and TE671) cell lines, expressed as fold increase over NSM, arbitrarily set at 1. GAPDH was used as control. Bars represent mean values of three independent experiments, each performed in triplicate. HFM, human foetal myoblats. **C.** Western blot showing the expression of DNMT3B protein in RH4, RH30, RD and TE671 cell lines. Tubulin was used as loading control. Representative of three different experiments.

To explore the association between DNA methylation and RMS, transcript levels of the *de novo* DNMT3B gene were assessed in 7 ARMS and 7 ERMS primary tumours by using Real Time PCR (Q-PCR). Expression of DNMT3B was significantly up-regulated in all tumour samples in comparison to NSM, used as normal tissue, with an average increase of 81.2±9.4 (Figure 1A). In accordance with previously published data [31], also DNMT1 mRNA levels were significantly higher in ARMS and ERMS tumours compared to NSM (30.5±9.0, data not shown). DNMT3A levels were also investigated, showing an up-regulation in RMS *vs.* NSM of 22.0±3.3 (data not shown). DNMT3B over-expression was also confirmed in ARMS (RH4 and RH30) and ERMS (RD and TE671) cell lines both at mRNA and protein levels (Figure 1B and 1C).

### siRNA transfection down-regulates DNMT3B expression and inhibits RMS cell proliferation

**Figure 2:**
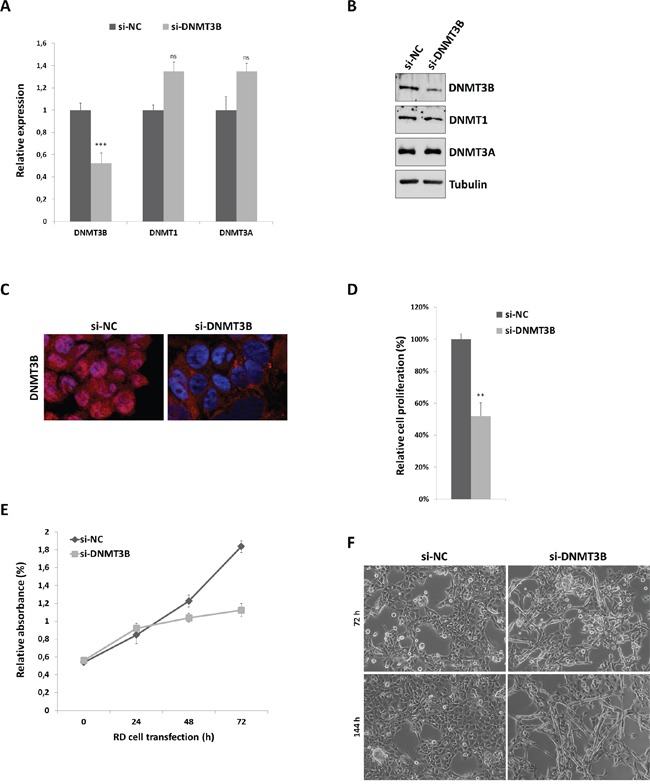
DNMT3B knock-down by RNA interfering and effects on RD cell viability and morphology **A.** DNMT3B transcript levels measured by Q-PCR at 72 h in RD cells after transfection with DNMT3B siRNA (si-DNMT3B) in comparison to samples transfected with non-targeting control siRNA (si-NC), arbitrarily set at 1. Specific transcript levels were normalized to GAPDH control mRNA. Shown are the means of four independent experiments. Error bars represent standard deviations of the means. Asterisks represent the statistical significance (***, p<0.001; ns, not significant). DNMT1 and DNMT3A mRNA levels were also analysed to check the specificity of siRNA-mediated silencing. **B.** Western blots showing the expression of DNMT3B protein at 72 h after si-DNMT3B transfection compared to si-NC cells. Tubulin was used as loading control. DNMT1 and DNMT3A protein levels were not perturbed by si-DNMT3B delivery. Representative of three independent experiments. **C.** Representative immunofluorescence showing the down-regulation of DNMT3B protein levels in nuclear compartment at 72 h after si-DNMT3B transfection. High levels of DNMT3B were evident in the nuclei of si-NC cells. **D.** Viability of RD cells 72 h post-transfection with DNMT3B siRNA calculated with respect to control si-NC cells, assessed by trypan blue exclusion staining. Results represents the mean value of four independent experiments ± SD. Statistical significance: **, p<0.01. **E.** MTT assay performed to assess relative RD cell numbers at different point after si-DNMT3B or si-NC transfection (0-24-48-72 h). Each point is the mean of three replicate wells ± SD and is representative of three independent experiments. **F.** Cellular morphology of si-NC and si-DNMT3B RD cells was analysed under light microscope at 20x magnification at 72 and 144 h after siRNA transfection. In si-DNMT3B cultures, more elongated cellular bodies were evident, many of which formed multinucleated myotube-like structures.

In order to evaluate the effect of DNMT3B enzyme on the phenotype of RMS cells, we used a specific small interfering RNA (siRNA) against DNMT3B mRNA in RD cell line, an *in vitro* model of ERMS. SiRNA transfections were performed in RD cells cultured in growth medium, i.e. supplemented with 10% serum. DNMT3B knock-down efficiency was assessed by using Q-PCR and western blot analysis at 72 h after transfection. A significant reduction of DNMT3B at both mRNA (0.4-fold) and protein (0.5-fold) levels (Figure 2A and 2B) was observed in si-DNMT3B cells compared to those transfected with the negative control siRNA (si-NC). Colorimetric assays confirmed that DNMT3B protein quantity was reduced about 0.5-fold after RNAi-specific silencing respect to mocked control (data not shown). DNMT1 and DNMT3A expression was not significantly perturbed by transient si-DNMT3B transfection, this assessing a specific silencing (Figure 2A and 2B). Moreover, immnunoflurescence experiments showed that the cellular distribution of DNMT3B was mainly into the nucleus in mocked control RD cells, whilst, upon si-DNMT3B transfection, a noticeable decline in its nuclear levels was detected (Figure 2C). At 72 h subsequent to transfection, direct counting for living cells using trypan blue dye exclusion test confirmed that DNMT3B depletion was able to drastically inhibit the proliferation potential of RD cells compared to si-NC cells (Figure 2D). Similar results were obtained by MTT assay, which showed a significant decrease of cellular viability/proliferation rate in si-DNMT3 compared to si-NC transfected cultures (Figure 2E). DNMT3B silenced cells exhibited a substantial change in their morphology with more elongated cellular bodies already after 72 h post-siRNA transfection (Figure 2F). At 144 h after transfection, si-DNMT3B cells showed even lower levels of DNMT3B itself (data not shown) and an increased number of multinucleated myotube-like structures (Figure 2F), whilst si-NC had high DNMT3B protein levels and showed the typical round proliferative shape (Figure 2F). DNMT3B siRNA transfection carried out in a second ERMS cell line (TE671) gave overlapping results with a marked reduction in DNMT3B protein levels in western blotting and immunofluorescence experiments (Supplementary Figure S1A-S1B). DNMT3B knocked-down TE671 cells also exhibited a minor cell growth rate (data not shown) and a more elongated morphology than mocked control cells (Supplementary Figure S1C) at both 72 and 144 h post-transfection.

### Decreased DNMT3B expression induces G1 cell cycle arrest and inhibits cell migration of RMS cells

**Figure 3:**
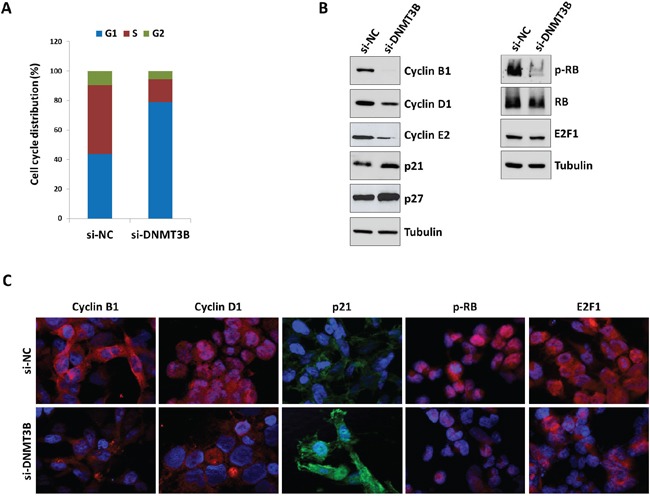
Depletion of DNMT3B levels induces G1 arrest and alters the expression of specific cell cycle regulators in RD cells **A.** Flow cytometry data showing percentages of cells in G1, S and G2 phases in si-DNMT3B and si-NC RD cells. Data are average values of three independent experiments. **B.** Western blot analyses of a panel of cell cycle regulatory proteins in RD cells at 72 h after si-NC or si-DNMT3B transfection. Tubulin expression was used as the internal control. Representative blots of three independent experiments. **C.** Immunofluorescence experiments showing the expression and localization of Cyclin B1, Cyclin D1, p21, p-RB and E2F1 proteins in RD cells at 72 h after DNMT3B or NC siRNA transfection. DAPI was used for nuclear staining. Images captured under ApoTome microscope at 40x magnification.

**Figure 4:**
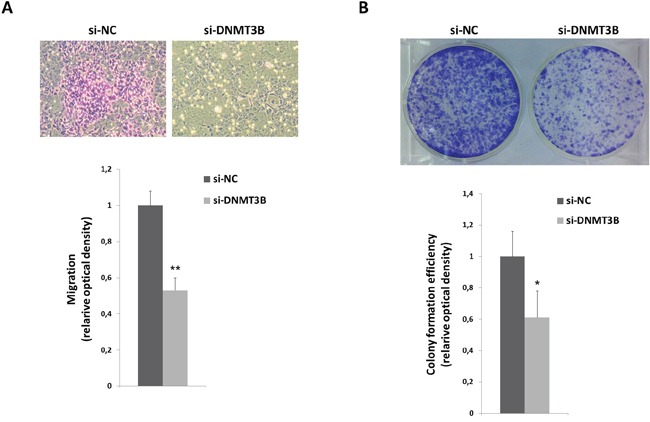
DNMT3B reduction correlates with altered migration and clonogenic ability of RD cells **A.** Representative images of RD si-DNMT3B and si-NC migrated cells using the transwell migration assay (magnification of 10x). Data in the histograms are expressed as the means ± SD from three separate experiments, each performed in triplicate (**, p<0.01 compared with the respective si-NC control, arbitrarily set at 1). **B.** RD cells transfected with si-DNMT3B or si-NC were seeded at low concentration and allowed to grow for 8 days to examine their colony formation capacity. Representative pictures of colonies stained with crystal violet. Colony forming efficiency was calculated by crystal violet absorbance from three independent experiments, each performed in triplicate (*, p<0.05 compared with the respective si-NC control, arbitrarily set at 1).

To further determine whether the reduced RD cell growth was due to alterations in cell cycle progression, flow cytometry analysis was performed. Based on propidium iodide staining of cellular DNA content, DNMT3B down-regulation resulted in a significant increase of cell percentage in G1 phase (p<0.005) with a concomitant decrease of cell percentage in S and G2 phases (G1: 78.0±2.8%, S: 15.1±2.5%, G2: 6.9±0.27%), whilst si-NC transfected cells rapidly divided and progressed through the cell cycle at high rates (G1: 43.5±0.7%, S: 44.9±2.0%, G2: 11.6±1.3%) (Figure 3A). Consistent with G1 arrest, the expression of different cell cycle regulators was modulated with a marked decrease of Cyclin B1, Cyclin D1 and Cyclin E2 expression (Figure 3B) and a simultaneous up-regulation of p21 and p27 levels in DNMT3B depleted cells (Figure 3B). As shown in Figure 3B, DNMT3B knowck-down also efficiently abolished the phosphorylation status of retinoblastoma (RB) tumour suppressor (p-RB) and induced a parallel slight reduction in E2F1 levels (0.2-fold), this confirming the cell cycle block at G1 phase. In accordance with Western blotting expression results, immunofluorescence experiments also showed that si-DNMT3B transfection caused a strong reduction of Cyclin D1 nuclear staining with a dramatic up-regulation and relocalization of p21 in the nuclear compartment (Figure 3C). Consistent with the RB activity in its underphosphorylated state, nuclear shuttling of the E2F1 transcriptional factor was not evident in si-DNMT3B cells compared to si-NC samples (Figure 3C). Programmed cell death was not affected by DNMT3B knock-down since no significant changes in early or late apoptosis rates were observed in si-DNMT3B compared to si-NC transfected cells, as assessed by flow cytometry experiments (data not shown). Furthermore, the expression levels of some specific apoptosis markers, such as cleaved PARP and BcL-Xl, resulted similar in DNMT3B-depleted cells and in negative control samples (data not shown). DNMT3B silencing significantly impaired the ability of RD cells to migrate through the non-matrigel-coated membranes towards serum-containing medium with respect to si-NC controls (Figure 4A). Finally, when allowed to grow at low density, si-DNMT3B cells showed a lower ability to form colonies in anchorage-dependent experiments compared to control cells, with a 0.6-fold of crystal violet absorbance (Figure 4B).

Together, these results indicated that the *in vitro* under-expression of DNMT3B is able to inhibit RMS progression by reducing cell proliferation and migration.

### DNMT3B knock-down promotes myogenic differentiation in RMS cells

**Figure 5:**
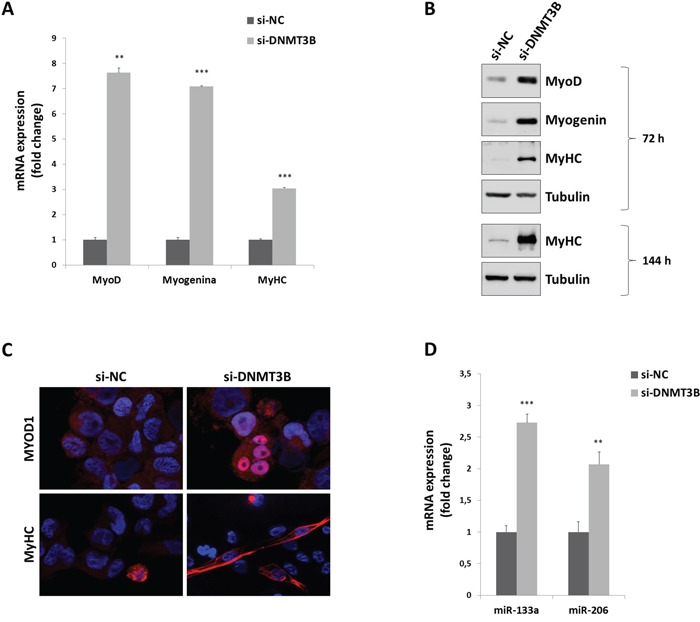
DNMT3B knock-down triggers myogenic differentiation in RD cells **A.** Expressionof myogenic genes, MYOD1, Myogenin and MyHC, by Q-PCR assays in RD cells transfected with DNMT3B or NC siRNAs for 72 h. Expression of each mRNA was normalized to GADPH levels and plotted as fold change relative to si-NC samples. Histograms are means ± SD. Asterisks represent the statistical significance (**, p<0.01; ***, p<0.001). **B.** Western blot showing the expression of MYOD1, Myogenin and MyHC proteins in si-DNMT3B and si-NC RD cells at 72-144 h post-transfection. Tubulin was used as loading control. Representative of three different experiments. **C.** Immunofluorescence experiments showing the expression and localization of MYOD1 and MyHC at 72 h after DNMT3B or NC siRNA transfection. DAPI was used for nuclear staining. Images captured under ApoTome microscope at 40x magnification. **D.** Expression of myogenic miRNAs, miR-133a and miR-206, by Q-PCR experiments in RD cells transfected with DNMT3B or NC siRNAs for 72 h. Expression of each miRNA was normalized to U6 levels and plotted as fold change relative to si-NC samples. Histograms are means ± SD. Asterisks represent the statistical significance (**, p<0.01; ***, p<0.001).

Since si-DNMT3B transfected cells showed a substantial change in their morphology with the acquisition of a myogenic-like phenotype, the expression of specific muscle markers was evaluated. A sustained increase of MYOD1, Myogenin and MyHC at mRNA and protein levels was observed in both Q-PCR and immunoblotting assays, performed 72 h after DNMT3B silencing respect to si-NC transfection (Figure 5A and 5B). In immunofluorescence experiments, MYOD1 factor exhibited a more evident granular staining in nuclear envelope in si-DNMT3B transfected RD cells in comparison to si-NC samples (Figure 5C); furthermore, elongated myotube-like si-DNMT3B cells displayed a strong fluorescence of MyHC, a marker of committed muscle cells (Figure 5C), indicating that the proper myogenic differentiation was triggered by the reduction of the DNMT3B levels. TE671 cells transfected with si-DNMT3B also showed up-regulation of MYOD1, Myogenin and MyHC protein levels at 72 h post-siRNA injection (Supplementary Figure S1D-S1E).

Notably, the phenotypic effect of DNMT3B knock-down in RD cells was also observed at 144 h post-transfection, with higher levels of MyHC protein (Figure 5B), confirming that the commitment to terminal myogenic differentiation was preserved. Finally, we investigated whether DNMT3B was also involved in the control of specific miRNAs. Indeed, we demonstrated that DNMT3B depletion was able to significantly up-regulate the expression of miR-133a and miR-206, two well-established myomiRs essential in promoting muscle cell differentiation (Figure 5E).

Taken together, these results show that the selective inhibition of DNMT3B gene can effectively promote a sustained myogenic program in ERMS cells, by inducing the proper temporal expression of myogenic genes and miRNAs important for differentiation.

### MEK/ERK inhibitor U0126 down-regulates DNMT3B protein expression

**Figure 6:**
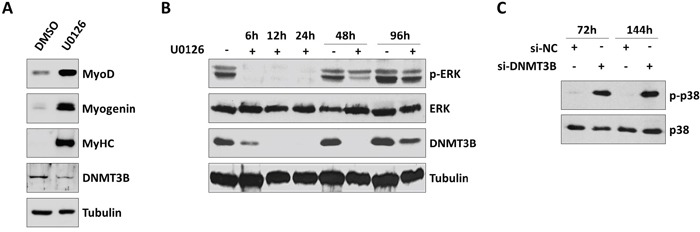
Inhibition of MEK/ERK pathway by U0126 down-regulates DNMT3B and induces myogenic program in RD cells **A.** Western blot showing the down-regulation of DNMT3B and the up-regulation of MYOD1, Myogenin and MyHC in RD cells treated with 10 μM U0126 for 96 h. Mocked control cells were treated with DMSO. Tubulin was used as loading control. Representative of three different assays. **B.** Time-course experiments showing the early decreased phosphorylation status of ERK (p-ERK) and the down-stream reduced expression of DNMT3B protein in RD cells upon U0126 treatment for 0-6-12-24-48-96 h. Tubulin was used as loading control. Representative of two different assays. **C.** Western blot showing phosphorylated and total p38 levels in si-DNMT3B and si-NC RD cells at 72 and 144 h post-transfection.

Since the MEK/ERK cascade is aberrantly activated in RMS and its inhibition has been shown to affect tumour growth and to rescue skeletal muscle differentiation in ERMS cells [38], as occurred in si-DNMT3B transfected cells, we studied if DNMT3B expression could be linked to the MEK/ERK signalling pathway. RD cells treated with the MEK/ERK inhibitor U0126 (10 μM) for 96 h were able to fully differentiate, as assessed by the up-regulation of MYOD1, Myogenin and MyHC levels, by impairing DNMT3B protein expression (Figure 6A). Interestingly, a drastic reduction of DNMT3B enzyme was observed at early points of U0126 treatment (12-24 h) in RD cells, subsequently to the down-regulation of ERK phosphorylation status, evident at 6 h post-exposure (Figure 6B). TE671 cells treated with U0126 showed matched results (Supplementary Figure S1F). Considering the evidence that U0126 treatment is able to activate p38 kinase for promoting myosin expression in RMS cells [38, 39], p38 phosphorylated levels were analysed in DNMT3B depleted cells. A long-lasting increase of p38 phosphorylation (p-p38) was observed in si-DNMT3B transfected cells in comparison to si-NC samples both at 72 than at 144 h in RD (Figure 6C) and TE671 cell lines (Supplementary Figure S1G).

Altogether, these data indicate that DNMT3B is a down-stream component of the MEK/ERK signalling pathway and that abrogation of this enzyme is an essential step in the U0126-mediated capacity of ERMS cells to rescue myogenic differentiation program.

## DISCUSSION

DNA hypermethylation, an epigenetic mechanism resulting in transcriptional silencing, is a common occurrence in tumour development [6, 40–42]. Aberrant methylation of CpG islands has also been observed in RMS [32, 43], a solid tumour characteristic of childhood and adolescence. Our study shows, for the first time, an aberrant expression of DNMT3B gene in both ARMS and ERMS primary tumours and cell lines. In particular, DNMT3B transcripts are over-expressed in RMS tumours compared to NSM, suggesting that high levels of this enzyme are important molecular features of RMS. Our results indicate that the inhibition of DNMT3B expression is a crucial step for reverting RMS cancer phenotype towards skeletal muscle differentiation, by restricting the expression of proliferative markers and up-regulating myogenic genes. Indeed, knock-down of DNMT3B gene by RNA interference impaired the *in vitro* tumour-promoting potential of ERMS cells by significantly reducing cell proliferation, migration and colony-forming ability. Consistent with the marked cellular growth arrest at G1/S-phase transition, altered levels of proteins involved in the cell cycle control were observed, supporting a key role of DNMT3B in sustaining RMS cell progression. One of the potential mechanisms that underline the DNMT3B-mediated arrest involves the ability of DNMT3B siRNA to reduce the expression of different cyclins (Cyclin B1, Cyclin D1 and Cyclin E2) and to block the pRB/E2F1 pathway by inducing de-phosphorylation of RB tumour suppressor. Indeed, hypophosphorylated RB represents the active form of the protein that is able to associate with E2F1 factor, hence preventing its nuclear translocation and blocking its ability to activate genes that are important in cell cycle progression [44]. The inhibition of cell proliferation was also confirmed at molecular levels by a significant increase in p21, whose up-regulation might be directly related to a promoter demethylation and a concomitant re-expression upon DNMT3B depletion. Interestingly, the efficient growth inhibition induced by DNMT3B knock-down was not associated to an increased apoptosis rate but to the restoration of myogenic fate in ERMS cell lines. The effect of DNMT3B *in vitro* down-regulation on the ARMS tumorigenic phenotype remains to be fully dissected, but preliminary results suggest that the differentiation program is not efficiently sustained in RH4 and RH30 cells. We can speculate that DNMT3B is an essential repressor of myogenic differentiation since forced DNMT3B down-regulation is able to activate muscle-regulatory factors (MRFs) and myomiRs in ERMS cell culture models, irrespective of high serum concentration levels in the culture medium. The observed decrease of DNMT3B protein levels upon treatment with U0126, an inhibitor of MEK/ERK signalling network, whose disruption has previously been demonstrated to reactivate myogenesis in ERMS cells [38, 39], further supports the hypothesis that DNMT3B expression must be turned off to enable a proper muscular differentiation. Compared to mocked control cells, si-DNMT3B samples showed of a marked expression of MYOD1, followed by Myogenin and myomiR (miR-133a and miR-206) up-regulation. MYOD1 is a master regulator of myogenesis, since it normally triggers the down-stream molecular events that switch myoblasts from a growing phase to a differentiated state, by directly promoting the expression of miR-206 and many other genes involved in myogenesis [45, 46]. RD proliferating cells show low levels of MYOD1, and increased abundance of this transcription factor has been demonstrated to fully execute the terminal myogenic program [47], this confirming that a controlled amount of MRFs is a fundamental condition for proper skeletal muscle differentiation in ERMS cells. Notably, DNMT3B silencing was maintained at 144 h after siRNA-transfection and determined long-term changes in ERMS cell phenotype, as assessed by the elevated levels of MyHC protein, a marker of terminal myogenic specification. Notably, DNMT3B knock-down induced a sustained activation of p38 kinase, which has been reported to promote myogenesis and to maintain myogenic-related morphology in ERMS cells [38, 39]. Based on the present results, DNMT3B may represent a new important molecule explaining why ERMS cells fail to activate/complete the skeletal muscle differentiation program and to exit the cell cycle. Therefore, understanding the DNMT3B-mediated molecular mechanisms affecting these pathways may yield further insights into pathophysiological processes in RMS tumours. Several studies have suggested that the methylation of the enhancer/promoter regions of muscle regulators is a critical event in the initiation of the myogenic transcriptional program [48]. Alterations of methylation levels occurring in the promoter of the human MYOD1 gene have been reported in RMS tumours, with partially methylated MYOD1 up-stream regions present essentially in ERMS subtypes [33]. Human MYOD1 gene is located at 11p15 [49], a chromosomal region containing the IGF2 and H19 imprinted genes [50], whose expression has been found to be altered (over-expression of IGF2 and downregulation of H19) in a subset of ERMS tumours [51]. Therefore, the abnormal MYOD1 methylation pattern observed in RMS might be due to a generalized deregulation of the 11p15 imprinted region. To this concern, over-expressed levels of the DNMT3B might be directly responsible for the epigenetic deregulation observed in RMS, since this enzyme is responsible of *de novo* DNA methylation patterns. DNMT3B might preserve skeletal muscle differentiation in ERMS cells by directly hypermethylating DNA or by cooperating with other factors able to modify chromatin, such as histone deacetylases (HDACs) and PcG proteins [26, 27, 52]. Previous reports have demonstrated that the activation/repression of muscle-related gene transcription is achieved by a coordinate sequence of epigenetic molecular mechanisms, comprehending both acetylation and methylation at specific 5′-flanking regulatory regions [7, 52, 53]. In line with this observations, exposure of RMS cells to demethylating agents or HDAC inhibitors has been shown to have pro-differentiative effects in RMS cells by up-regulating the expression levels of different muscle-specific coding genes and miRNAs [34, 35, 54]. Transcriptional repression by hypermethylation of both DNA and histones at the promoter/enhancer regions of myogenic factors and cofactors has recently been proposed as a potential mechanism to promote tumour formation and progression as well as to impair terminal differentiation in RMS [55–57]. Recent findings have shown that DNMT3 enzymes can repress transcription in a methylation-independent manner [58], since their capacity to bind silencer complexes, comprehending MBD proteins, or specific transcriptional repressors [25, 59–61]. Indeed, in addition to the highly conserved DNMT catalytic domain at C-terminus [62], DNMT3 family proteins have at N-terminus a plant homeodomain-like motif, which is present in many chromatin-associated proteins and mediates protein-protein interactions [63]. So, the molecular mechanisms of DNMT3B-mediated transcriptional silencing at muscle-gene regulatory regions in RMS could be due to its potential repressor activity. Preliminary results indicate that DNMT3B and MYC are able to interact in RD cells (Megiorni & Marampon, unpublished data), suggesting a possible molecular mechanism for site-specific DNA methylation/repression of target genes in ERMS tumours. Bisulfite sequencing and chromatin immunoprecipitation assays are currently underway in our laboratory to characterize the possible epigenetic modifications at enhancer/promoter regions associated with MYOD1, Myogenin and MyHC expression in si-DNMT3B RMS cells in comparison to control mocked samples. Understanding the molecular mechanisms associated with the activated muscular differentiation after DNMT3B depletion is essential to design novel pharmacological treatments for this soft tissue sarcoma. Future studies will also be aimed at globally characterising the methylome profile in si-DNMT3B cells in order to provide a deeper insight into the RMS tumorigenesis and to identify new potential targets for the development of more effective and less toxic epigenetic therapies. Indeed, 5-aza and 5-aza-dC DNA demethylanting agents have been approved by US Food and Drug Administration for clinical use but, despite some promising results in laboratory models and in haematological malignancies [64, 65], their efficacy in the treatment of human solid tumours is still controversial [66]. Furthermore, these drugs are two nucleoside analogues that are incorporated into nucleic acids so that long-term exposure induces high levels of toxicity [67]. Non-nucleoside compounds, which are independent of replication for their incorporation into DNA, or other molecules with a high biochemical selectivity towards individual human DNMT enzymes might lead to promising anticancer therapies [68, 69], and this also in RMS.

In summary, this study describes for the first time the over-expression of DNMT3B gene in RMS primary tumours and cell lines, suggesting that an aberrant transcriptional control mediated by this *de novo* DNMT is a key event in RMS development and progression. Altogether our findings also outline the potential clinical application of DNMT3B inhibition in cancer treatment. DNMT3B target therapies may represent a novel clinical approach, effective not only in impairing tumour growth but also in reactivating myogenic program, especially in ERMS subtypes.

## MATERIALS AND METHODS

### Patient clinical features

Fourteen RMS primary tumour samples, 7 ARMSs and 7 ERMSs, were obtained at diagnosis before any treatment from children admitted to the Department of Oncology at the Alder Hey Children's NHS Foundation Trust, Liverpool. Histopathological diagnosis was confirmed using immunohistochemistry. All 7 ARMS cases were investigated for PAX3/7-FOXO1 translocations using standard FISH analysis: 5 tumours were PAX3-FOXO1-positive, 1 was PAX7-FOXO1-positive and 1 was fusion-negative. Patients were grouped according to the Intergroup Rhabdomyosarcoma Study (IRS) postsurgical grouping system [70]. Details of the patients have previously been described [35]. Institutional written informed consent was obtained from the patient's parents or legal guardians. The study underwent ethical review and approval according to the local institutional guidelines (Alder Hey Children's NHS Foundation Trust Ethics Committee, approval number 09/H1002/88). Control RNA was extracted from normal skeletal muscle (NSM) obtained from eight children undergoing surgery for non-oncological conditions.

### RNA isolation

Total RNA was extracted using TRIzol (Invitrogen, Carlsbad, CA) according to the manufacturer's instructions. RNA quality and quantity were checked using NanoDrop 2000 (Thermo Scientific, Waltham, MA).

### Cell lines and cultures

Alveolar (RH4 and RH30) and embryonal (RD and TE671) cells were maintained in high-glucose Dulbecco's modified Eagle's medium supplemented with 10% foetal bovine serum (FBS), 1% v/v L-glutamine, 100 μg/ml streptomycin and 100 U/ml penicillin, and grown at 37°C in a humidified atmosphere of 5% CO_2_. To minimize the risk of working with misidentified and/or contaminated cell lines, we later stocked the cells used in this report at very low passages and used at <20 subcultures. DNA profiling using the GenePrint 10 System (Promega Corporation, Madison, WI) was carried out to authenticate cell cultures, comparing the DNA profile of our cell cultures with those found in GenBank.

### Quantitative real time PCR (Q-PCR)

Total RNA (2 μg) was subjected to reverse transcription (RT) with High Capacity cDNA Reverse Transcription kit (Applied Biosystems, Foster City, CA) according to the manufacturer's instructions. Quantitative Real Time PCR (Q-PCR) for human DNMT1 (Hs00154749_m1), DNMT3A (Hs01027166_m1), DNMT3B (Hs00171876_m1) and MyHC (Hs00428600_m1) mRNAs was performed using specific TaqMan Real-Time Gene Expression Assays (Applied Biosystems). Q-PCRs for human MYOD and Myogenin transcripts was carried out with the SensiFAST SYBR Hi-ROX Kit (Bioline, London, UK). Primer sequences are available under request. All Q-PCR assays were performed on a StepOne Real Time System (Applied Biosystems) machine. Samples were normalized according to the glyceraldehyde-3-phosphate dehydrogenase (GAPDH) mRNA levels. RT for human miR-133a (000458) and miR-206 (000510) was carried out with TaqMan MicroRNA Assay kit (Applied Biosystems) using 20 ng of total RNA sample and the specific stem-loop primer according to manufacturer's protocols. Data were normalized to U6 small nuclear RNA (RNU6, 001093) levels. The amount of each mRNA/miRNA was calculated by the comparative Ct method and expressed as fold change using the StepOne v2.3 software (Applied Biosystems). Each sample was run in triplicate in at least three independent experiments, unless specified differently in the figures.

### Transient transfection

RD and TE671 cells were seeded at 4×10^5^ cells/well in 6-well plates; small interfering RNA (siRNA) against human DNMT3B (si-DNMT3B, sc-37759 by Santa Cruz Biotechnology, Dallas, TX) or siRNA negative control (si-NC, sc-37007 by Santa Cruz Biotechnology) were combined with RNAiMAX (Invitrogen) and used at 60 nM final concentration; si-DNMT3B is a pool of 3 target specific 19-25 nt siRNAs designed to specifically knock-down DNMT3B gene and this product was previously validated in other publications [41, 71]. Cells were collected for cell cycle analysis, Q-PCR, western blot, immunofluorescence, migration, *etc.* at different times after transfection. All the experiments were performed in proliferating growth medium, i.e. supplemented with 10% FBS.

### DNMT3B colorimetric quantification

The EpiQuik Dnmt3B Assay Kit (Epigentek, Farmingdale, NY) was used to colorimetrically measure the amount of DNMT3B in nuclear extracts of si-NC and si-DNMT3B RD cells after 72 h post-transfection, according to the manufacturer's instructions. Experiments were repeated on RD cells after two independent transfections.

### Cell proliferation assays

Direct counting of RD and TE671 living cells was performed with trypan blue exclusion dye (Sigma-Aldrich, Saint Louis, MO) using the Countess II Automated Cell Counter (Thermo Fisher) at 72 h after si-DNMT3B or si-NC transfection. Experiments were repeated at least three times. Cell viability of the siRNA-transfected cells was determined using MTT [3-(4,5-dimethylthiazol-2-yl)-2,5-diphenyltetrazolium] assay. RD cells (10^4^) were plated onto 96-well plates in sextuplicates and, after 24 h, transfected with synthetic DNMT3B siRNA; mocked control cells (transfected with si-NC) and blank cell-free wells were also included. At designated times after transfection (0-24-48-72 h), 10 μl of MTT (5 mg/ml, Sigma-Aldrich) were added to each well and plates were incubated at 37°C for 3 h. Media were removed and 200 μl of DMSO was added into each well to dissolve the dark blue formazan crystals. Absorbance was read at wavelength of 550 nm, with reference at 630 nm, using a microtitre plate reader (SelectScience, Corston, UK). The percentage cellular viability was calculated with the appropriate controls taken into account. The results were plotted as means ± SD of two separate experiments having six determinations per assay for each experimental condition.

### Morphological assessment of siRNA-transfected cells

An Axio Vert.A1 microscope (Carl Zeiss Microscopy, Thornwood, NY), furnished with an AxioCam MRc5 camera (Carl Zeiss Microscopy) was used to observe the morphological changes of the cells transfected with si-DNMT3B or si-NC siRNAs. Cells were photographed at 72 and 144 h post-transfection using a 20x magnification. Images were analysed by using the ImageJ software (NIH ImageJ 1.47).

### Cell cycle analysis

RD and TE671 cells transfected with DNMT3B or NC siRNAs were counted at 72 h post-transfection and at least 10^6^ cells were fixed in 70% ice-cold ethanol overnight at +4°C. Cell pellets were washed twice with ice-cold PBS and treated with RNase A (50 μg/ml) for 30 min at 37°C. Propidium iodide (10 μg/ml) was added to each sample and DNA content was measured by BD FACSCalibur Flow Cytometer (BD Biosciences, San Jose, CA). Data were analysed using ModFit 3.1 software (BD Biosciences). Experiments were carried out three times.

### Trans-well migration assay

For the cell migration assay, BD FalconTM Cell Culture Inserts with 8 μm pore polycarbonate filters were placed into a 24-well culture plate. Briefly, serum-free cell suspension containing 10^5^ si-DNMT3B or si-NC RD cells was added to the upper compartment of the chamber at 48 h post-transfection. The lower compartment contained DMEM-HG with 10% FBS, used as chemoattractant. After incubation at 37°C for additional 24 h, migrated cells at the base of the inserts were fixed in 100% methanol and stained with 1% crystal violet dye. Non-migrating cells on the upper surface of the membrane were removed with cotton swabs. Cells were photographed under a light microscope at 10x. The area occupied by migrated cells in si-DNMT3B and si-NC samples was measured by using the ImageJ software (http://imagej.nih.gov/ij/). Experiments were carried out three times.

### Colony formation assay

For anchorage-dependent colony formation assays, si-DNMT3B or si-NC transfected RD cells were plated in 6-well plates at 4×10^3^ cells/well and cultured for 8 days. Colonies were fixed in 100% methanol and stained with 1% crystal violet dye in methanol and photographed. To quantify colonies, crystal violet was solubilized in 30% acetic acid in water for 15 min at RT and absorbance was measured using the Biochrom Libra S22 UV/VIS spectrophotometer (Biochrom, Berlin, DE) at wavelength of 595 nm; 30% acetic acid in water was used as the blank. Colony formation capacity in si-DNMT3B cells in comparison to si-NC samples was calculated as follow: (OD_si-NC_ - OD_si-DNMT3B_/OD_si-NC_) x 100%, having three determinations per experiment. Assays were carried out three times.

### U0126 treatment

RD and TE671 cells were seeded at 4×10^5^ cells/well in 6-well plates. After 24 h, the MEK/ERK inhibitor U0126 (Santa Cruz Biotechnology) was added to a final concentration of 10 μM. Following different times of treatment, cells were collected for western blot experiments. Mocked control cells were treated with DMSO.

### Protein extracts and western blot analysis

RD and TE671 cells, transfected with siRNAs or treated with U0126, were lysed in RIPA buffer, as previously described [72]. Experiments were performed at different times after siRNA transfection or drug treatment. Total protein extracts (30 μg) were separated on 8-12 % sodium dodecyl sulfate (SDS)-polyacrylamide gel and transferred onto polyvinylidene fluoride (PVDF) membranes (EMD Millipore Corporation, Billerica, MA). Filters were blocked in 5 % not-fat milk or BSA and incubated over-night at +4°C with the following primary antibodies: Bcl-XL, Cyclin B1, Cyclin E2, DNMT3B, E2F1, p21 and p27 by Santa Cruz Biotechnology; cleaved PARP, MYOD1, Myogenin and MyHC by EMD Millipore Corporation; DNMT1 and DNMT3A by GeneTex, Irvine, CA; Cyclin D1 by MBL International, Woburn, MA; phospho-pRB at Ser807/811 (p-RB), total RB, phospho-p38 at Thr180/Tyr182 and total p38 by Cell Signalling Technology, Danvers, MA. Appropriate horseradish peroxidase (HRP)-conjugated secondary antibodies (Santa Cruz Biotechnology) were used for 1 h at RT. Protein signals were detected using WesternBright ECL kit (Advansta, Menlo Park, CA), according to the manufacturer's instructions, and visualized by ChemiDoc XRS+ (Bio-Rad). Tubulin (Sigma-Aldrich) was used as a normalization control for equal loading. Densitometry was performed to quantify changes in protein expression using the Image Lab 5.1 software (Bio-Rad). Briefly, signal intensity for each band was calculated by the local subtraction method. DNMT3B protein levels in si-DNMT3B samples were then normalized and reported as relative expression with respect to si-NC cells. All experiments were carried out at least three times.

### Immunofluorescence

Immunofluorescence experiments were performed as previously described [72]. Briefly, RD and TE671 cells transfected with DNMT3B or NC siRNAs were cultured for 48 h before plating 10^5^ cells per well in 24-well plates with 2% gelatine-coated glasses. After one additional day, cells were fixed in 4% paraformaldehyde for 30 min and incubated with the following primary antibodies: Cyclin B1, DNMT3B, E2F1 and p21 (Santa Cruz Biotechnology); MYOD1 and MyHC (EMD Millipore Corporation); Cyclin D1 (MBL International); p-RB (Cell Signalling Technology). Appropriate Texas Red-coniugated secondary antibodies (Jackson ImmunoResearch, West Grove, PA,) were used. Nuclei were counter-stained with 1 μg/ml 4′, 6-diamido-2-phenylindole dihydrochloride (DAPI, Sigma-Aldrich). Labelled cover-slips mounted in Moviol were acquired with Zeiss ApoTome and Axiovision software (Carl Zeiss) using a 40x magnification. Experiments were replicated twice.

### Statistical analysis

Data were expressed as mean ± standard deviation (SD) of each condition. Statistical significance between groups was assessed by Student's t-test and probability (p) values of less than 0.05 were accepted as significant.

## SUPPLEMENTARY FIGURE


