# Supplementary material for: DNMT3B in vitro knocking-down is able to reverse embryonal rhabdomyosarcoma cell phenotype through inhibition of proliferation and induction of myogenic differentiation
Source: Oncotarget. 2016 Oct 15;7(48):79342–56. doi: 10.18632/oncotarget.12688 (PMC5346718; doi:10.18632/oncotarget.12688)
Supplement: Supplementary file 1 [file oncotarget-07-79342-s001.pdf]

DNMT3B *in vitro* knocking-down is able to reverse embryonal rhabdomyosarcoma cell phenotype through inhibition of proliferation and induction of myogenic differentiation

SUPPLEMENTARY FIGURE

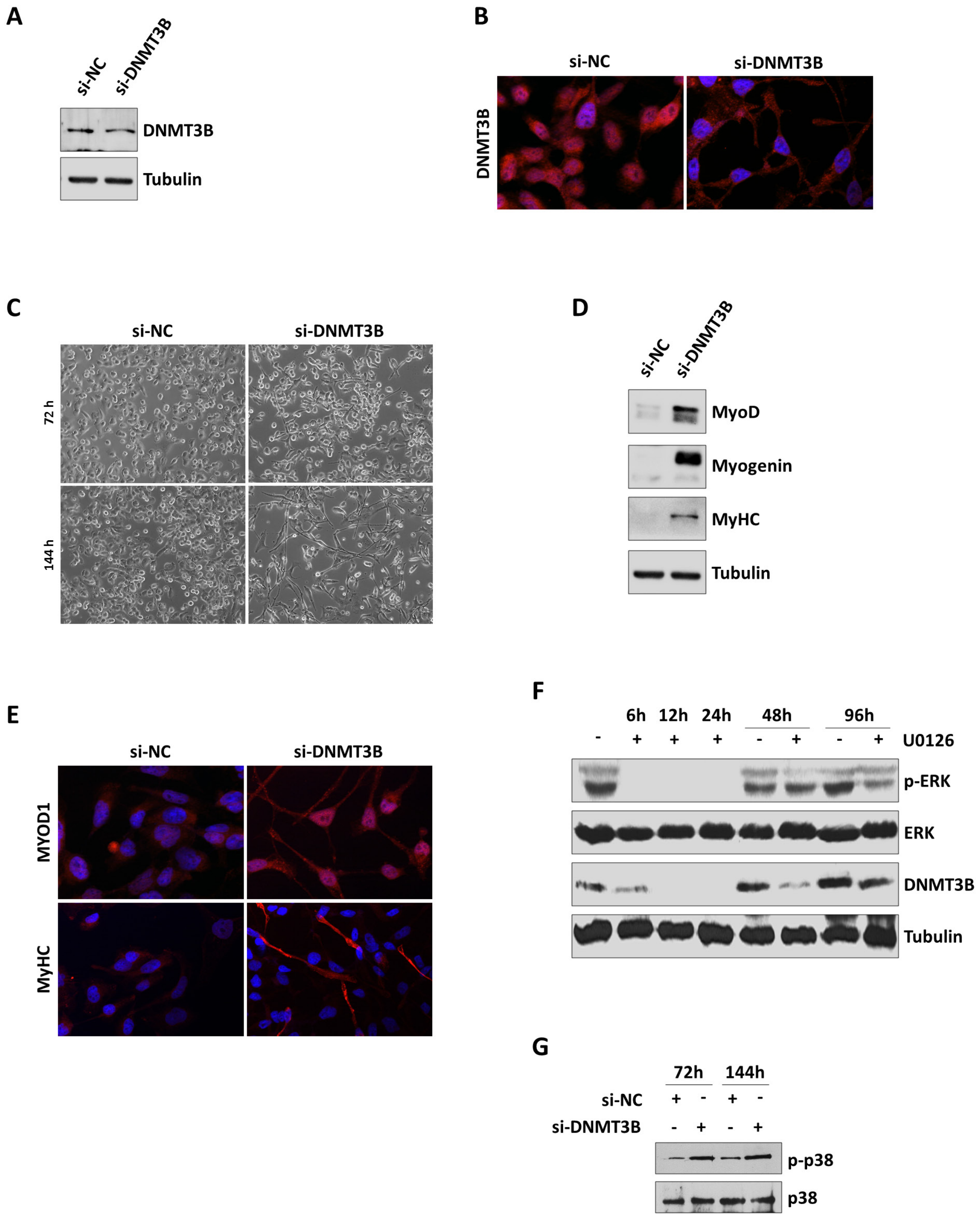

(*Continued*)

Supplementary Figure S1: DNMT3B knock-down by RNA interfering induces terminal myogenic differentiation in TE671 cells. A. Western blots showing the expression of DNMT3B protein at 72 h after si-DNMT3B transfection compared to si-NC cells. **B.** Representative immunofluorescence showing the down-regulation of DNMT3B protein levels in nuclear compartment at 72 h after si-DNMT3B transfection. High levels of DNMT3B were evident in the nuclei of si-NC cells. **C.** Cellular morphology of si-NC and si-DNMT3B TE671 cells was analysed under light microscope at 20x magnification at 72 and 144 h after siRNA transfection. In si-DNMT3B cultures, more elongated cellular bodies were evident, many of which formed multinucleated myotube-like structures. **D.** Western blot showing the expression of MYOD1, Myogenin and MyHC proteins in si-DNMT3B and si-NC TE671 cells at 72 h post-transfection. Tubulin was used as loading control. Representative of three different experiments. **E.** Immunofluorescence experiments showing the expression and localization of MYOD1 and MyHC at 72 h after DNMT3B or NC siRNA transfection. DAPI was used for nuclear staining. Images captured under ApoTome microscope at 40x magnification. **F.** Time-course experiments showing the early decreased phosphorylation status of ERK (p-ERK) and the down-stream reduced expression of DNMT3B protein in TE671 cells upon U0126 treatment for 0-6-12-24-48-96 h. Tubulin was used as loading control. Representative of two different assays. **G.** Western blot showing phosphorylated and total p38 levels in si-DNMT3B and si-NC TE671 cells at 72 and 144 h post-transfection.
